# Estimated Cerebrospina Fluid Pressure and the 5-Year Incidence of Primary Open-Angle Glaucoma in a Chinese Population

**DOI:** 10.1371/journal.pone.0162862

**Published:** 2016-09-09

**Authors:** Lan Li, Cairui Li, Hua Zhong, Yijin Tao, Yuansheng Yuan, Chen-Wei Pan

**Affiliations:** 1 Department of Ophthalmology, the First Hospital of Kunming, Kunming, China; 2 Affiliated Hospital of Dali University, Dali, China; 3 Department of Ophthalmology, the First Affiliated Hospital of Kunming Medical University, Kunming, China; 4 Jiangsu Key Laboratory of Preventive and Translational Medicine for Geriatric Diseases, School of Public Health, Medical College of Soochow University, Suzhou, China; National Eye Institute, UNITED STATES

## Abstract

**Purpose:**

We aim to assess the longitudinal association between baseline estimated cerebrospinal fluid pressure (CSFP) and 5-year incident primary open angle glaucoma (POAG) in a population-based sample of Bai Chinese living in rural China.

**Methods:**

Among the 2133 Bai Chinese aged 50 years or older who had participated in the baseline examination of the Yunnan Minority Eye Study, 1520 (71.3%) attended the follow-up examination after five years and 1485 were at risk of developing POAG. Participants underwent comprehensive ophthalmic examinations at both baseline and follow-up surveys. CSFP in mmHg was estimated as 0.55 × body mass index (kg/m2) + 0.16 × diastolic blood pressure (mmHg)-0.18 × age (years)-1.91. Glaucoma was defined using the International Society of Geographical and Epidemiological Ophthalmology Classification criteria. Multivariate logistic regression models were established to determine the association between baseline CSFP and incident POAG.

**Results:**

After a mean follow-up time of 5 years, 19 new cases of POAG were detected, with an incidence rate of 1.3% (95% confidence interval, 0.7–1.9%). In multivariate logistic regression analysis, after adjusting for age, gender, education, intraocular pressure, central corneal thickness, hypertension and diabetes, no significant associations, nor any trends, were evident between baseline estimated CSFP and incident POAG. The association between estimated CSFP per mmHg increase in baseline and 5-year incidence of POAG was also non-significant, with adjusted relative risk of 0.96 (P = 0.11) in multivariate analysis.

**Conclusions:**

This longitudinal cohort study does not support previously observed cross-sectional association between estimated CSFP and POAG in population-based studies.

## Introduction

Glaucoma is the leading cause of irreversible blindness throughout the world, which affects more than 60 million people currently and will affect more 100 million by the year 2040.[1] Primary open angle glaucoma (POAG) is the most predominant subtype of glaucoma among general populations.[2–5] Thus, understanding the pathophysiology of POAG is crucial for reducing the global burden of blindness and improving people’s vision-related quality of life.

Although quite a few clinical variables such as enlarged cup-to-disc ratio (CDR), CDR asymmetry, elevated intraocular pressure (IOP) and thinner central corneal thickness (CCT) have been well established to be the clinical risk factors for POAG, their absence does not effectively rule out POAG.[6] Recent studies indicated that cerebrospinal fluid pressure (CSFP) or trans-lamina cribrosa pressure difference may be involved in the pathogenesis of glaucomatous optic neuropathy.[7–10] In a retrospective clinical study on patients who had undergone lumbar CSFP measurements, patients with POAG and without glaucomatous optic neuropathy were compared and the authors found that the lumbar CSFP was significantly lower in the POAG group than in the non-glaucomatous group.[11] Several population-based studies including the Korean National Health and Nutrition Examination Survey[12], the Beijing Eye Study[13], and the Central India Eye and Medical Study[14] found that lower CSFP or higher trans-lamina cribrosa pressure difference was related to a higher prevalence of POAG. However, most of the studies were retrospective or cross-sectional in nature, from which the evidence is far from conclusive. In addition, the association observed previously was low in magnitude and the biological plausibility remains unclear. Selection bias inherent to the case-control design and the lack of temporal information provided by the cross-sectional nature of population-based surveys has made the findings less convincing. Population-based longitudinal assessment would not only reduce the selection bias of the studied population but also be important for clarifying the potential causal association by providing temporal information. To the best of our knowledge, there have been no population-based longitudinal studies reporting the association between CSFP and incident POAG.

In the present study, we aim to assess the longitudinal association between baseline estimated CSFP and 5-year incident POAG in a population-based sample of Bai Chinese living in rural China.

## Methods

### Study population

The Yunnan Minority Eye Study (YMES) is a population-based eye study among ethnic minorities in Yunnan Province in southwest China. The baseline examination for the Bai Chinese was conducted in 2010 and the 5-year follow-up examination in 2015. The study methods for the baseline and follow-up exanimation have been described in previous reports.[15–17] In brief, random cluster sampling strategies were adopted to select ethnic Bai adults aged 50 years or older living in rural communities at baseline examinations. Each village in the study site with a population of approximately 1000 was considered as a cluster during the sampling procedure. Villages with a population of less than 750 were combined and those of more than 1500 were divided and regrouped. Subsequently, 10% of the total clusters were randomly selected using a computer-assisted program. An adult was considered “ineligible” to participate if he or she had moved from the residing address, had not been living there for more than 6 months, or was deceased. A total of 2133 participants (77.8%) were examined in the baseline survey. All subjects in the baseline survey were invited to the 5-year follow-up examination and similar examination procedures were used for the baseline and follow-up studies. For the subjects who did not attend examination site, home visits or revisits were conducted if necessary. Nonparticipants in the follow-up study were those who had moved away without updated contact information, who refused to participate, or who died before the commencement of the 5-year follow-up examination. Death information was confirmed through village death registration forms. For the current data analysis, participants who had received a diagnosis of definite glaucoma at baseline were excluded. All the participants included in the incidence study were free of glaucoma at baseline.

Our study was approved by the Kunming Medical University Ethics Review Board. It was conducted in accordance with the tenets of the World Medical Association Declaration of Helsinki after written informed consent was obtained from all the participants.

### Clinical examinations

The clinical examination methods used in follow-up study were the same as those in the baseline survey, which were described in detail previously.[16] Distance visual acuity (VA) was measured with a 4-m logarithm of the minimum angle of resolution tumbling E chart, including uncorrected and best-corrected VA. Slit-lamp biomicroscopy examination (Model SL-1E; Topcon, Tokyo, Japan) was performed to examine the external and anterior segment of the eye including eyelid, globe, and pupillary reflex. The vertical cup-disc ratio (VCDR) was measured by direct ophthalmoscopy and slit-lamp biomicroscopy with a 90-D convex lens without pupil dilation. VCDR and minimal neural rim widths were the key indicators of glaucomatous optic structural changes, and the point of maximum inflection of the vessels crossing the neuroretinal rim under stereoscopic view was defined as the cup margins of VCDR. Digital fundus photographs focusing on the optic disc were taken using a retinal fundus camera (APS-A; KangHua CO., LTD, Chongqing, China) and were subsequently assessed through stereo viewers. IOP was initially measured with a handheld tonometer (TONO-PEN AVIA; Reichert Inc., New York, NY) after instilling 1 drop of oxybuprocaine hydrochloride (0.4% Benoxil; Oxybuprocaine, Santen, Japan). IOP readings from the Goldmann Applanation Tonometry (Zeiss AT 030 Applanation Tonometer; Carl Zeiss, Jena, Germany) were considered as the final measurement and were performed for all glaucoma suspects. The CCT was measured for both eyes using an ultrasound pachymeter (Advent; Mentor O & O Inc, Norwell, Massachusetts) and the mean of the 5 readings was used for data analysis. Non-cycloplegic autorefraction was performed using an autorefractor (RM-8000; Topcon Corp., Tokyo, Japan). Spherical equivalent (SE) was defined as sphere plus half cylinder and myopia was defined as SE of less than -0.5 diopter (D).

### Definitions of glaucoma

Study participants with any of the following signs were considered as glaucoma suspects: VCDR ≥ 0.7 in either eye, VCDR asymmetry ≥ 0.2, neuroretinal rim width reduced to < 0.1 CDR (between 11 and 1 o’clock or 5 and 7 o’clock), IOP >21 mm Hg, optic disc hemorrhage, notch and nerve fiber layer defect. All glaucoma suspects were asked to return for visual field (VF) testing on a designated day. White-on-white automated perimetry (Humphrey 750; Carl Zeiss Meditec, Inc., Dublin, CA) was performed with near refractive correction. The SITA-Fast 24–2 mode was used throughout.

Glaucoma cases were defined according to the International Society for Geographical and Epidemiological Ophthalmology (ISGEO) criteria based on 3 categories.[18] Category 1 cases were defined as optic disc abnormality (VCDR or VCDR asymmetry ≥ 97.5^th^ percentile or neuroretinal rim width from the 11- to 1-o’clock position or the 5- to 7-o’clock position < 0.1 VCDR) and glaucomatous visual field defect. Category 2 cases were defined as a severely damaged optic disc (VCDR or VCDR asymmetry ≥ 99.5th percentile) in the absence of an adequate visual field test. When diagnosing category 1 or 2 glaucoma, it was required that there be no other explanation for the VCDR finding (ie, dysplastic disc or marked anisometropia) or visual field defect (ie, retinal vascular disease, macular degeneration, or cerebrovascular diseases). Category 3 cases were defined as blindness in individuals who had no visual field or optic disc data (corrected visual acuity, <3/60) and who had undergone previous glaucoma surgery or had an IOP greater than the 99.5^th^ percentile. POAG was defined as an eye with evidence of glaucoma (as defined in this paragraph) with an angle appearance in which the posterior trabecular meshwork was seen for 180° or more of the angle circumference during gonioscopy. Final identification, adjudication, and classification of glaucoma cases were made by a glaucoma specialist (Hua Zhong). Study participants were defined to have incident POAG if they did not have POAG at baseline but had POAG in either eye at the follow-up visit.

### Measurement of estimated cerebrospinal fluid pressure and other variables

Participants’ educational level, socioeconomic status, lifestyle-related factors, disease history, and medication intake was collected using a questionnaire. Height was measured in centimeters using a wall-mounted measuring tape after removing shoes. Weight was measured in kilograms after taking off heavy clothing. Body mass index (BMI) was calculated as the weight in kilograms divided by the square of the height in meters. Systolic blood pressure, diastolic blood pressure and pulse rate for all participants were recorded using a standardized mercuric-column sphygmomanometer, and one of four cuff sizes (pediatric, regular adult, large, or thigh) was selected based on the circumference of the participant’s arm.

Using a previously developed formula[12], CSFP in mmHg was estimated as 0.55 × BMI (kg/m^2^) + 0.16 × diastolic blood pressure (mmHg)-0.18 × age (years)-1.91. This estimation has been used in quite a few population-based studies and has shown to be highly correlated with lumbar CSFP measurements.

### Statistical analysis

Data analyses were performed using commercial software (SPSS, version 16.0; SPSS, Inc). Multivariate logistic regression models were established to determine the association between baseline CSFP and incident POAG. Incident POAG was analyzed as the dependent variable and baseline CSFP as the independent variable. Age, gender, education, IOP, CCT, hypertension and diabetes were considered as covariates and were adjusted in the model. We examined variables of interest in univariate models first. Only age, sex, factors with a p value of less than 0.05 in univariate analyses or factors of scientific importance were considered in multivariate analyses. The interactions between CSFP and other variables with incident POAG were determined using a likelihood ratio test. Relative Risks (RRs) and 95% confidence intervals (CI) are presented.

## Results

Among all 2133 participants who had participated in the baseline examination, 1520 successfully attended the follow-up examination in 2015, representing for a 71.3% follow-up rate of the original cohort. The mean age of the study participants in follow-up survey was 63.5 ± 8.3 years (range: 55–95 years). Among the other 613 adults who did not participated in the follow-up survey in 2015, 248 (11.6%) were dead and 365 (17.1%) did not agree to be re-examined or had moved away. Table 1 compares the baseline characteristics among participants who returned at the 5-year examination and those who did not. Compared with nonparticipants, the study participants were younger, heavier, less likely to have diabetes, and had higher estimated CSFP at baseline. The baseline estimated CSFP were 11.7±1.1 mmHg among adults who participated in the follow-up examination.

**Table 1:** Comparison of baseline characteristics among participants seen in the follow-up examination and those died or did not return. Data presented are means (standard deviations) or number (%), as appropriate for variable.

	Participants seen at 5-year exam (n = 1520)		Participants died or did not return (n = 613)		P
Age (years)	63.5	8.3	67.4	9.2	<0.001
Height (cm)	158.6	8.5	157.4	15.4	0.15
Weight (kg)	55.5	9.7	53.1	10.3	0.02
Sex (female)	961	63.2	403	65.7	0.26
Education (no formal)	507	33.4	252	41.1	<0.001
Hypertension	788	51.8	297	48.4	0.33
Diabetes	28	1.8	20	3.3	0.01
Intraocular pressure (mmHg)	14.5	3	14.4	3.1	0.65
Central corneal thickness (um)	536.3	24.7	536.5	22.2	0.32
Estimated cerebrospinal fluid pressure (mmHg)	11.7	1.1	10.7	1.1	0.03

Of the 1520 participants in the follow-up examination, 35 participants who had received a diagnosis of definite glaucoma at baseline were excluded from the current analyses. Table 2 shows the association of baseline estimated CSFP and incident POAG in this cohort. Univariate analyses were performed in the first step. In univariate analyses, baseline IOP and hypertension were significantly related to incident POAG. In multivariate analysis, after adjusting for age, gender, education, IOP, CCT, hypertension and diabetes, no significant associations, nor any trends, were evident between baseline estimated CSFP and incident POAG (all P > 0.05). The association between estimated CSFP per mmHg increase in baseline and 5-year incidence of POAG was also non-significant, with adjusted RR of 0.96 (CI 0.84–1.19) in multivariate analysis (Table 2). The explanatory variables included in the multivariate model accounted for 68% of the total variations in the incidence of POAG in this population (R^2^ = 68%). No interaction effects were observed (all P > 0.05).

**Table 2:** Association of estimated cerebrospinal fluid pressure at baseline and incident POAG. POAG = Primary Open-Angle Glaucoma; CI = Confidence Interval.

Estimated cerebrospinal fluid pressure at baseline	Persons at risk	Incident POAG cases	Age-gender-adjusted model			Multivariate-adjusted model*		
			Relative Risk	95%CI	P	Relative Risk	95%CI	P
First quartile	371	6	1.9	0.4–3.6	0.56	1.9	0.3–3.9	0.74
Second quartile	372	5	1.8	0.7–5.2	0.63	1.8	0.7–5.4	0.69
Third quartile	371	5	1.7	0.7–5.6	0.35	1.5	0.6–5.8	0.53
Fourth quartile	371	3	Reference group			Reference group		
Per mmHg increase	1485	19	0.95	0.75–1.08	0.09	0.96	0.84–1.09	0.11

*Adjusted for age, gender, education, intraocular pressure, central corneal thickness, hypertension and diabetes

We also analyzed the association between changes in estimated CSFP within 5 years and the incidence of POAG and the results are presented in Table 3. Changes in estimated CSFP within 5 years were not associated with the incidence of POAG.

**Table 3:** Association of changes in estimated cerebrospinal fluid pressure within 5 years and incident POAG. POAG = Primary Open-Angle Glaucoma; CI = Confidence Interval.

Changes in estimated cerebrospinal fluid pressure within 5 years	Persons at risk	Incident POAG cases	Age-gender-adjusted model			Multivariate-adjusted model		
			Relative Risk	95%CI	P	Relative Risk	95%CI	P
First quartile	371	6	1.2	0.8–2.5	0.43	1.1	0.8–2.5	0.60
Second quartile	371	5	1.1	0.7–3.2	0.55	1.1	0.7–3.3	0.50
Third quartile	372	4	1.1	0.7–3.4	0.63	1.0	0.7–3.6	0.55
Fourth quartile	371	4	Reference group			Reference group		
per mmHg increase	1485	19	0.87	0.55–1.28	0.22	0.90	0.53–1.41	0.46

*Adjusted for age, gender, education, intraocular pressure, central corneal thickness, hypertension and diabetes

## Discussion

Although the role of CSFP in glaucomatous pathophysiology is beginning to emerge, scarce available population-based longitudinal data have greatly limited our understanding of this possible contributory mechanism in POAG development. Our study is the first population-based longitudinal analysis on the association between estimated CSFP and the incidence of POAG and fills the gap of knowledge in this area.

Previous studies have reported a weak and cross-sectional association between CSFP or trans-laminar pressure difference and the prevalence of POAG among general populations and the evidence supporting this hypothesis has been summarized in a meta-analysis.[19] However, this cross-sectional association is not supported by the current longitudinal analysis in our study. Using the YMES 5-year follow-up data, we did not find any significant association or trend between the baseline estimated CSFP and the incidence of POAG. Findings from our study suggest that there may not be a real and statistically significant association between CSFP and POAG. A possible association found between increasing CSFP as a continuous variable and incident POAG in our study indicates that any true association would likely be very weak, if it existed. Alternatively, such a finding could have resulted from incomplete adjustment for potential confounding factors, or may be just a chance finding. Current data is not enough to support the role of CSFP in the development of POAG.

Although several biological mechanisms have been raised to explain the association between CSFP or trans-laminar pressure difference and POAG[8, 20, 21], the exact mechanisms are still unclear. An animal study on monkeys explored the association between low CSFP and glaucomatous optic nerve damage. Four monkeys received a lumbar-peritoneal shunt to reduce CSFP and the other five were treated as controls. Chronic reduction in CSFP associated with an optic neuropathy in some monkeys. However, it was unclear whether the optic nerve damage was glaucoma-like. [22] Chowdhury et al [23]established a comprehensive animal model where CSFP can be manually controlled over an extended period of time, which is important in understanding the role of CSFP in the development of glaucomatous optic neuropathy. However, there are quite a few issues that need to be addressed regarding the potential role of CSFP in glaucoma. As CSFP dynamics are poorly understood, it is unclear whether cardiac variations in CSFP exist or how body positions or activities of the individuals could influence it. Although IOP diurnal fluctuations are greater in eyes with glaucoma[24], it needs to be clarified whether there are similar variations in CSFP and single measurement of CSFP can represent short or long-term pressure variations that could have a role in glaucoma.

From an epidemiologic perspective, a longitudinal cohort design provides information on antecedent exposures and consequent outcomes and the evidence from longitudinal cohort studies is usually considered to be much more cogent than that from case-control or cross-sectional designs. But survival bias is a major concern in longitudinal cohort designs. In our study, we found that those who died over the follow-up period were more likely to have lower estimated CSFP at baseline (Table 1). If a true association between lower CSFPs and higher risks of POAG exists, the persons who had died during the follow-up period would have been more likely to develop POAG if they were still alive. Therefore, our non-significant finding of the association between CSFP and incident POAG could have resulted from survival bias.

Although our study is the first longitudinal study in this research area, potential limitations should also be acknowledged. First, the whole study depended on the estimation of CSFP being derived from a multivariate formula incorporating BMI, diastolic blood pressure and age. The result of this formula was then termed CSFP and correlated with incident POAG. Although the estimated CSFP was primarily just the result of a mathematical equation, the calculated CSFP values correlated well with invasively measured CSFP values in previous reports. Second, the incidence of POAG in this cohort is not high and statistical power for detecting a significant association may not be enough. Last but not least, survival bias or lost-to-follow-up bias could have distorted the associations as mentioned previously.

## Conclusions

In summary, the longitudinal YMES data do not support previously observed cross-sectional association between estimated CSFP and POAG in population-based studies. Overall, longitudinal findings from this study suggest that it is still far from conclusive to suggest that CSFP could predict the development of POAG. However, survival bias could have influenced the longitudinal association and the direction of the bias would more likely be towards null in our study population.
